# Supplementary material for: Alzheimer’s Disease blood biomarkers measured through remote capillary sampling correlate with cognition in older adults
Source: Nat Commun. 2026 May 6;17:3699. doi: 10.1038/s41467-026-71448-2 (PMC13149973; doi:10.1038/s41467-026-71448-2)
Supplement: Supplementary file 1 — Reporting Summary [file 41467_2026_71448_MOESM1_ESM.pdf]

nature portfolio

Corresponding author(s): Anne Corbett

Last updated by author(s): 2026/02/17

Reporting Summary

Nature Portfolio wishes to improve the reproducibility of the work that we publish. This form provides structure for consistency and transparency in reporting. For further information on Nature Portfolio policies, see our Editorial Policies and the Editorial Policy Checklist.

Statistics

For all statistical analyses, confirm that the following items are present in the figure legend, table legend, main text, or Methods section.

n/a	Confirmed	
☐	☒	The exact sample size (*n*) for each experimental group/condition, given as a discrete number and unit of measurement
☐	☒	A statement on whether measurements were taken from distinct samples or whether the same sample was measured repeatedly
☐	☒	The statistical test(s) used AND whether they are one- or two-sided<br>*Only common tests should be described solely by name; describe more complex techniques in the Methods section.*
☐	☒	A description of all covariates tested
☐	☒	A description of any assumptions or corrections, such as tests of normality and adjustment for multiple comparisons
☐	☒	A full description of the statistical parameters including central tendency (e.g. means) or other basic estimates (e.g. regression coefficient) AND variation (e.g. standard deviation) or associated estimates of uncertainty (e.g. confidence intervals)
☐	☒	For null hypothesis testing, the test statistic (e.g. *F*, *t*, *r*) with confidence intervals, effect sizes, degrees of freedom and *P* value noted<br>*Give P values as exact values whenever suitable.*
☒	☐	For Bayesian analysis, information on the choice of priors and Markov chain Monte Carlo settings
☒	☐	For hierarchical and complex designs, identification of the appropriate level for tests and full reporting of outcomes
☐	☒	Estimates of effect sizes (e.g. Cohen's *d*, Pearson's *r*), indicating how they were calculated

Our web collection on statistics for biologists contains articles on many of the points above.

Software and code

Policy information about availability of computer code

Data collection	No software was used
Data analysis	Analysis was performed using IBM SPSS Statistics (Version 29) and R statistical software (Version 4.4.3).

For manuscripts utilizing custom algorithms or software that are central to the research but not yet described in published literature, software must be made available to editors and reviewers. We strongly encourage code deposition in a community repository (e.g. GitHub). See the Nature Portfolio guidelines for submitting code & software for further information.

Data

Policy information about availability of data

All manuscripts must include a data availability statement. This statement should provide the following information, where applicable:

- Accession codes, unique identifiers, or web links for publicly available datasets
- A description of any restrictions on data availability
- For clinical datasets or third party data, please ensure that the statement adheres to our policy

The anonymised data supporting this analysis are available on request from the PROTECT Research Group at the University of Exeter following approval by contacting data.protect@exeter.ac.uk

Research involving human participants, their data, or biological material

Policy information about studies with human participants or human data. See also policy information about sex, gender (identity/presentation), and sexual orientation and race, ethnicity and racism.

Reporting on sex and gender	This analysis applied to both sexes and the analysis controls for sex. Sex was self-reported by participants as part of online demographics collection
Reporting on race, ethnicity, or other socially relevant groupings	No socially relevant categorisation was conducted in this study
Population characteristics	This research included adults aged 65 and over in two cohorts. One cohort included participants with dementia who fulfilled criteria for mild to moderate dementia based on the National Institute of Neurological and Communicative Disorders and Stroke and Alzheimer's Disease and Related Disorders Association (NINCDS-ADRDA) criteria with impairment in at least two cognitive domains with impact on functionality. The second comparison cohort were cognitively healthy participants
Recruitment	Participants with dementia were recruited through primary and secondary care sites across South-West UK. Cognitively healthy participants were recruited through the nationwide PROTECT-UK ageing cohort using established online and digital protocols.
Ethics oversight	National Health Service (NHS) Research Ethics Committees (NHS REC London Bridge, Ref 13/LO/1578; NHS REC Greater Manchester East, Ref: 24/NW/0151).

Note that full information on the approval of the study protocol must also be provided in the manuscript.

Field-specific reporting

Please select the one below that is the best fit for your research. If you are not sure, read the appropriate sections before making your selection.

[x] Life sciences [ ] Behavioural & social sciences [ ] Ecological, evolutionary & environmental sciences

For a reference copy of the document with all sections, see nature.com/documents/nr-reporting-summary-flat.pdf

Life sciences study design

All studies must disclose on these points even when the disclosure is negative.

Sample size	A sample size of 150 participants was selected to provide sufficient clinical validity in cognitive data to allow for comparison and correlative analyses
Data exclusions	No data was excluded from this analysis
Replication	Analysis was independently conducted by two investigators to confirm outcomes
Randomization	N/A
Blinding	N/A

Reporting for specific materials, systems and methods

We require information from authors about some types of materials, experimental systems and methods used in many studies. Here, indicate whether each material, system or method listed is relevant to your study. If you are not sure if a list item applies to your research, read the appropriate section before selecting a response.

Materials & experimental systems

n/a	Involved in the study	
[x]	[ ]	Antibodies
[x]	[ ]	Eukaryotic cell lines
[x]	[ ]	Palaeontology and archaeology
[x]	[ ]	Animals and other organisms
[x]	[ ]	Clinical data
[x]	[ ]	Dual use research of concern
[x]	[ ]	Plants

Methods

n/a	Involved in the study	
[x]	[ ]	ChIP-seq
[x]	[ ]	Flow cytometry
[x]	[ ]	MRI-based neuroimaging

Plants

Seed stocks	N/A
Novel plant genotypes	N/A
Authentication	N/A
